# “It is something that gives us hope”: Lived experience among parents to children with cerebral palsy who are non-ambulant of the phenomenon physical activity, with or without the use of a novel dynamic standing device

**DOI:** 10.3389/fresc.2023.1139847

**Published:** 2023-04-24

**Authors:** Katarina Lauruschkus, Robert Holmberg, Åsa B. Tornberg

**Affiliations:** ^1^ Child and Family Health Research Group, Department of Health Sciences, Faculty of Medicine, Lund University, Lund, Sweden; ^2^ Division of Work and Organizational Psychology, Department of Psychology, Faculty of Social Sciences, Lund University, Lund, Sweden

**Keywords:** children, cerebral palsy, GMFCS level IV-V, parents, lived experience, participation, physical activity, dynamic standing

## Abstract

**Introduction:**

Regular physical activity confers health benefits for all. Parents commonly want their children to be physically active, and want to be physically active themselves, but children with cerebral palsy (CP) who are non-ambulant face challenges, and they need support to be physically active. Dynamic standing in the novel motorized assistive device Innowalk has positive effects in children who are non-ambulant—it gives them a chance to be physically active. The aim of this study was to explore the lived experience of physical activity of parents themselves and for their children with cerebral palsy who are non-ambulant.

**Methods:**

A descriptive inductive design with a hermeneutic phenomenological approach was used for the analysis of interviews with 11 parents of children with CP who are non-ambulant who participated in a study of exercise effects of dynamic standing.

**Results:**

The parents experienced physical activity for their children as being important but difficult, especially for their child, as described in Theme 1: “Being aware of health benefits while struggling with family time.” The children were perceived as being dependent on other people, the environment, and equipment for participating in physical activity, referring to Theme 2: “Being dependent.” The opportunity for their children to become physically active on a regular basis through an assistive device gave the parents hope for a better life, which formed Theme 3: “Getting hope in a challenging life situation.”

**Conclusion:**

Physical activity for children with CP who are non-ambulant is possible through an elaborate network of social relations and environmental conditions. Limiting the degree of dependence and containing the negative consequences of high a degree of dependence are vital in the support of physical activity. Relations, support, and assistive devices that strengthen empowerment and autonomy should be prioritized, and if this works, the experience of physical activity can be positive, giving families hope.

## 1. Introduction

It is common knowledge that regular physical activity in combination with minimal sedentary time confers health benefits for all. The international physical activity recommendations include people with disabilities, and adults are recommended to be physically active for at least 150 min weekly and children for 60 min daily, both with a limitation of sedentary time (1–3). Moreover, physical activity is a fundamental right for all, according to the United Nations Educational, Scientific and Cultural Organization (UNESCO). The right to physical activity can be linked to the Universal Declaration of Human Rights, to the Convention on the Rights of the Child, and to the Convention on the Rights of Persons with Disabilities (4). Many people follow the physical activity recommendations; others do not. Parents commonly expect and want their children to be physically active and want to be physically active themselves. Physical activity may be experienced as being positive for health and participation. On the other hand, the recommendations can be hard to achieve, and parents’ expectations may cause stress and they may experience it as their failure and being bad parents if their child doesn't reach the recommendations (5).

Children with cerebral palsy (CP) spend most of their time being sedentary, and they face challenges in reaching the physical activity recommendations that children without CP do not face (6, 7). Both frequency and intensity of physical activity decrease with increased severity of limitations in motor and cognitive function (7–9). CP is the most common physical disability in childhood, with an estimated prevalence in high income countries of 1.6 per 1,000 children (10). CP is often accompanied by conditions such as intellectual, communication, and behavioral impairment, as well as epilepsy and pain (11, 12). For children with CP who are non-ambulant—meaning that they cannot walk or sit without support—their gross motor function is classified as level IV or V according to the five-level Gross Motor Function Classification System Expanded and Revised (GMFCS-E&R), where level V implies the most severe gross motor function limitations (13). When asked, children with CP say that they want to participate in self-selected physical activities with the right support (14). Parents desired friends for their child with CP and competent adults to facilitate participation in physical activities (15).

**Figure 1:**
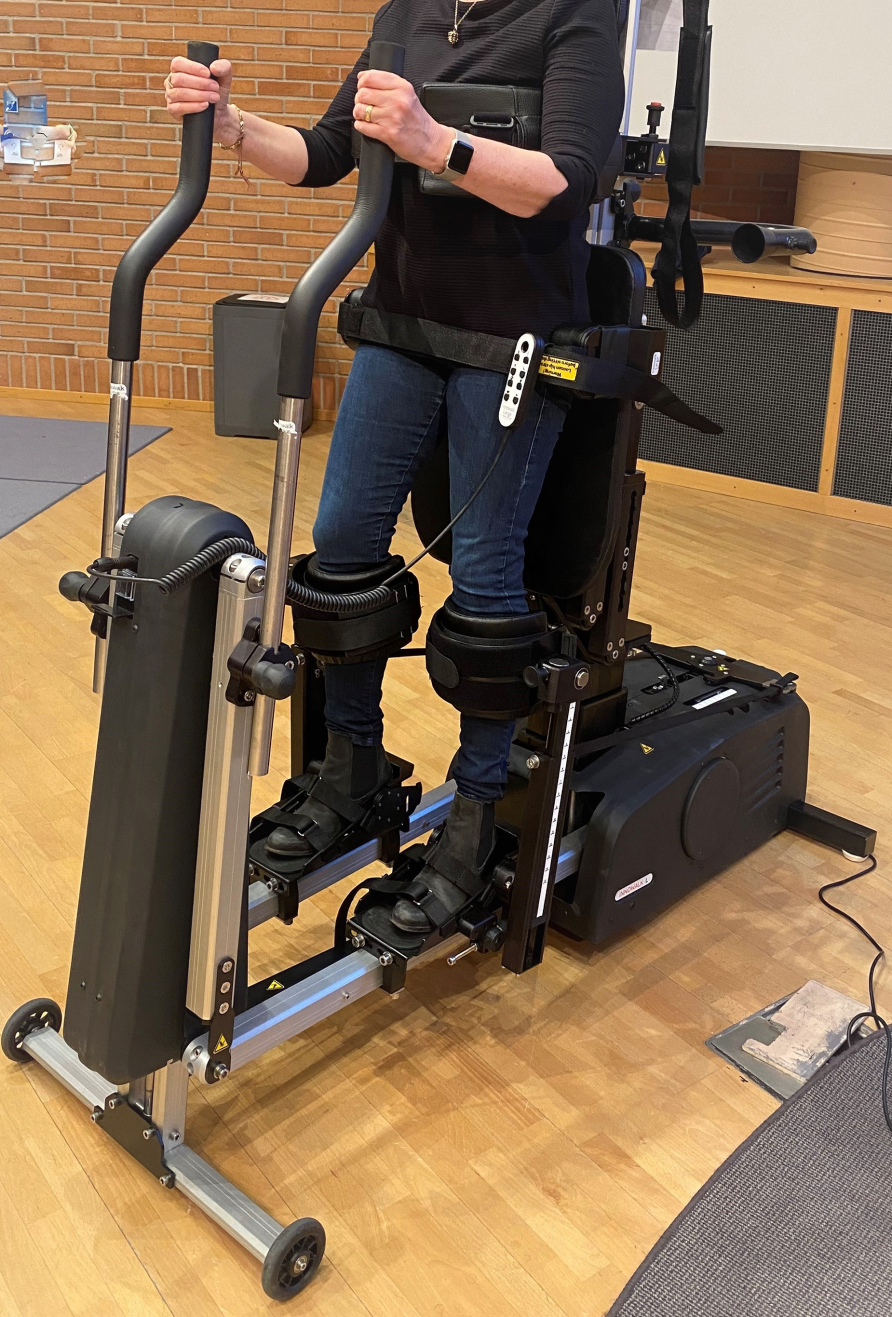
Dynamic standing exercise in the Innowalk.

The standard care in Sweden for children with CP who are non-ambulant includes daily static supported standing for 30–90 min. Supported standing is not passive for those who are non-ambulant and can be described as light physical activity, reducing sedentary behavior. In addition, supported standing is recommended not only for improved bone mineral density, passive range of motion, and reduced spasticity, but also to promote active movement, position change, and fitness (16–18). However, static standing in various types of standers allows no lower body movements, and while many children experience wellbeing being in an upright position, others experience inconvenience and pain. Therefore, we compared 4 months daily static with 4 months daily dynamic standing exercise for 20 children with CP who are non-ambulant. Dynamic standing exercise was enabled by the Innowalk, a novel motorized assistive device (Made for Movement, Norway) (Figure 1). The Innowalk offers whole-body exercise through assisted and repetitive walking movements of the lower extremities in an upright weight-bearing position, with the possibility to move or rest the arms. It is not a walker, and Innowalk is like a cross-trainer for people who cannot stand without support. The people exercising in the Innowalk do not actively take steps; it is a robotic device that performs the movement at individually chosen speed. However, dynamic standing in the Innowalk has shown to be a physical activity and is not passive. We found that dynamic standing had positive effects regarding the passive range of motion and spasticity in the hip, on bowel function, on pain, and on health-related quality of life (19, 20). Another study, of 46 patients, found that dynamic standing exercise in the Innowalk was secure to perform and improved passive assisted motion, intestinal function, body stability, and joint mobility (21).

Introducing new devices to support physical activity for children who are non-ambulant involves a network of people including the child, caregiver and other adults important for the child, assistants, healthcare professionals, and technicians. A new assistive device and the associated training regimes and practices will necessarily bring with them a need for change and learning at the level of the individual, the family, and at the interface between families and healthcare professionals as well as in the organizations providing services. Feasibility studies and implementation research aim to provide knowledge that can inform development, testing, and implementation of new innovations, methods, and treatments (22). Caregivers and health professionals have to adapt and act with good sense in specific situations, but most studies describe general strengths, weaknesses, barriers, and facilitators. We, therefore, argue that studies of physical activity and new devices have to better represent the complexity of phenomena in order to provide inspiration and guidance for users of research in new and complementary ways. In this study, we, therefore, aimed to take this approach further by going beyond general patterns and instead capture the lifeworld of the family with a focus on the meaning of physical activity. Descriptions that capture the lifeworld of the central actors—the child and their closest caregivers, their parents—has the potential to empower all involved actors to proceed with thoughtful action: “action full of thought and thought full of action” (23, p. 159). An inquiry guided by a hermeneutic phenomenological interest into the unique lived experience can add significantly to extant research and can help equip both parents and healthcare professionals with an attitude of curiosity, openness, and understanding that will help them through processes of change, learning, and adaptation. Therefore, the aim of this study was to gain knowledge of the lived experience of the physical activity phenomenon for the parents themselves and for their child with CP who is non-ambulant. The lived experience among parents of their child's physical activity was explored with or without the use of a novel dynamic standing device, as the children need extensive support to be physically active.

## 2. Materials and methods

### 2.1. Design

A descriptive inductive design with a hermeneutic phenomenological approach based on van Manen’s study (23) was used to describe parents’ lived experience. Hermeneutic phenomenology is a descriptive methodology where lived experience is recollected after it is passed or lived through. The thinking behind the hermeneutic phenomenological approach is to go beyond what is said and to understand the underlying experience of what has been lived through. van Manen (23) used four basic existential sentiments to describe this: “lived body,” “lived space,” “lived time,” and “lived human relations.”

### 2.2. Setting

Interviews were planned to be performed in the families’ homes or at a place the interview participants chose, but restrictions due to the Covid-19 pandemic intended that all interviews were performed on the virtual platform Zoom. Zoom is a collaborative, cloud-based videoconferencing service offering features including online meetings, and secure recording of sessions (Zoom Video Communications Inc., San José, CA, United States).

### 2.3. Participants

All parents of the 14 children who had been research participants in a study on the long-term exercise effects of dynamic standing for non-ambulant children with CP [ClinicalTrial.gov (LU2019LEER)] were invited to be interviewed. According to the study protocol, 4 months, three times weekly, of high-intensity interval training and 4 months, three times weekly, of moderate-intensity continuous training with a 4-month washout period should be completed. Due to the Covid-19 restrictions, some changes needed to be done and some participants continued their exercise period longer than planned and did not change the training regime. The dynamic standing exercise was performed in the children's home directly supervised and controlled by a parent or a personal assistant. The inclusion criteria for the exercise study were non-ambulatory children with CP, aged 10–17 years, and living in the Skåne Region in Sweden. All the 11 parents who agreed to participate in this interview study were primary caregivers. Ten of them were biological parents and one was a foster parent, eight were mothers and three were fathers, and five were born outside Europe. In two interviews, both parents were present, which made a total of 11 parents to 9 children. The children were aged 10–17 years, and seven were female. They had CP, GMFCS levels IV (*n* = 6) and V (*n* = 3), spastic CP (*n* = 7) or dyskinetic CP (*n* = 2), and mild (*n* = 3) or moderate-to-severe (*n* = 6) intellectual disability.

### 2.4. Data collection

KL interviewed all participants from June 2020 to April 2021, utilizing a semi-structured interview guide developed by the research team prior to data collection. First, a few minutes were used talking about physical activity in general. Then, the participants were asked to talk about what physical activity meant to themselves and to their child and, in addition, what physical activity meant for their own and for their child's wellbeing. The next question was about physical activity for a child with and/or without disability. The participants were asked which factors contributed to their child being physically active or inactive with regard to the International Classification of Functioning, Disability, and Health's personal and environmental factors (24). The participants were then asked to talk about their experiences with the dynamic standing exercise in the Innowalk. During the interviews, additional questions were asked, intended to encourage further and more reflective narration, such as: “Can you tell me more about this?”, “Can you tell me more about how this felt?”, or “Can you give me an example?” The interviews were audio-recorded and KL transcribed all interviews verbatim. The interviews lasted 25–47 min.

### 2.5. Data analysis

Our approach to analysis was informed by the hermeneutic phenomenological methodology outlined by van Manen (23). As a first stage, we read the transcripts with the aim to code all meaning units related to physical activity along four existential structures of the lifeworld: lived space, lived body, lived other, and lived time (23). KL coded all interviews while RH and ÅBT independently coded half of the interviews each. We worked in parallel with two to three interviews and had four meetings during which we compared coding and discussed the texts, with the aim to arrive at a consensus. After all interviews had been coded, KL created an Excel file with all meaning units. In-between meetings, we also wrote notes about the interviews and observations and reflections occasioned by the reading. In the second stage, we worked collaboratively in meetings in which we thematized the codes regarding how they were related to physical activity. We discussed the validity of the suggestions for possible themes and used the Excel file to go back to codes and search for alternative interpretations. The third stage in our analysis took the form of sharing and reflecting on the texts on which we based our work in the earlier stages, and we aimed to represent the phenomenon of physical activity in the lifeworld of parents and their children.

### 2.6. Preunderstanding

KL had a longstanding experience, and ÅBT and RH had some experience, of working with children with disabilities or chronic diseases. During the whole process, from planning and conducting the interviews through to all stages of the analysis, the authors reflected on their preunderstanding. To assure trustworthiness, quotations are used in the Results section.

### 2.7. Ethical considerations

The study was performed according to the World Medical Association Declaration of Helsinki (25) and was approved by the Swedish Ethical Review Authority (Reg. no. 2019-00106). The parents gave their informed consent in writing before the interviews took place. Parents were guaranteed confidentiality and the right to discontinue the interview at any time, and they agreed to the interviews being audio-recorded.

## 3. Results

The parents experienced being physically active as important with many health benefits but difficult, especially for their child with CP, as described in Theme 1: “Being aware of health benefits while struggling with family time.” The child was perceived by the parents as being dependent on people, environment, and equipment for participating in physical activity, referring to Theme 2: “Being dependent.” The opportunity for their children to become physically active on a regular basis through an assistive device gave the parents hope for a better life, which formed Theme 3 “Getting hope in a challenging life situation.” The themes and subthemes are presented in Table 1. None of the parents experienced negative effects of physical activity in general or of being physically active in the Innowalk.

**Table 1:** Themes and subthemes.

Being aware of health benefits while struggling with family time	Being dependent	Getting hope in a challenging life situation
Promoting health benefits	Being dependent on others	Living near life and death
The care of the child being a burden	Being dependent on an accessible environment	Getting hope
	Being dependent on access to assistive devices	

### 3.1. Being aware of health benefits while struggling with family time

Parents described being physically active as important for the whole family, for themselves as parents, for siblings, grandparents, and for their child with CP. The parents experienced their own physical, psychological, and mental health benefits from physical activity, and they found similar effects for their child. However, they were struggling with getting time and energy for everything that needed to be done for the child.

#### 3.1.1. Promoting health benefits

Being physically active was experienced as leading to improved wellbeing and major health benefits.

The parents described themselves as being happy, gaining energy, and getting a good feeling in the body from physical activity. When parents got around to exercise, they expressed having more energy to support their child.

Horseback riding, which is very physical, gives me mental recharge, I am reloading. There is nothing else that gives me this feeling of presence, it is totally here and now, and it gives me the balance of getting tired in the brain and in the body. That is what you want to achieve …. you feel fresher in your head and calmer in your body. (Mother to child 7)

It is natural, and it feels important to try to keep the body a little alert and strong. With [my daughter], it is this thing of being able to lift her and so on, so keeping the body moving and exercising it is quite important to be able to cope with heavy lifting and so on, and also to cope mentally as well. (Mother to child 12)

They were aware that physical activity was even more important for their child.

That are some of the things one should, you always should do more of … so do I think of those things when you are physically active that you are actually doing it. [I] prioritize gymnastics, [I] prioritize horseback riding and this gymnastic program for her lungs. [I] really need to ensure that these things are getting done, they are really important for her. (Mother to child 1)

You become calmer in the soul … when you have done something. And so it is for him, you notice it, he becomes calmer. And then he sleeps better, he has moved around a bit … Then he gets warmer in the body. Otherwise, he is … he is freezing cold, but when he stomps [exercises in the Innowalk], he gets warmer. That is the blood circulation. It is because the feet are cold otherwise. When he has stepped on them, then they become …, you can really feel that when you touch them afterwards … You can tell that they are getting warmer … he feels good when he exercises. (Father to child 10)

If she has many impressions, she gets tired in her head, now she gets tired in the whole body. That is the difference. (Mother to child 14)

#### 3.1.2. The care of the child being a burden

The parents narrated that they constantly had a bad conscience from not being or doing enough. They wanted to support their child in being physically active while there were many other activities and chores that needed to be done with and for the child.

Right now, it feels really heavy actually, both mentally heavy not being able to offer her so many fun options anymore, and then it's physically heavy because she's big and very, very stiff. (Mother to child 7)

At the same time, siblings could call for attention, and the parents’ own recovery must wait. The parents could not always prioritize being physically active themselves. All chores, medical issues, and the enormous number of necessary contacts were time-consuming and perceived as a burden affecting the whole family.

There is a lot of lifting and there is a lot of care, so it will be quite a lot … yes. Almost all [our] physical activity is included there. It is … if we are doing gymnastics with him and so, yes, that is our physical activity. (Father to child 10)

In addition, the parents’ energy levels were not always high enough to support their child in being physically active.

It is crucial that we have assistants, and time, time and motivation. So it is not so much that is needed, it is just me, just now it is my fault, but it will become better next time. It is not so much; one just has to take time for that and try to motivate her. (Mother to child 14)

Parents said that their own energy level often differed from their child's energy level, both when the child had a high or low energy level, and this difference was experienced as challenging.

### 3.2. Being dependent

The parents said that participation in physical activity was only possible through extensive support—the child could not be independently physically active but needed support for every physical activity however small. The child was dependent on people, on the environment, and on tailored and individually adjusted assistive devices to be able to participate in physical activity.

#### 3.2.1. Being dependent on others

The parents felt a strong need for individual support and professional competence when their child wanted to be physically active. They described their children as being completely dependent on them and others. The parents themselves experienced dependence on others for support and help for their child.

And sometimes it can happen, if we have assistants who come and go, there can be errors or delays. She consumes assistants because she has a really challenging mood which often causes trouble, and then … yes, yes you understand. And all together means it can be very chaotic. (Mother to child 6)

Having the right people around was described as being of the utmost importance for a sense of stability and consistency in their life situation. People around their child were personal assistants, school assistants, teachers, and other important adults.

It can be the person lifting her, if you lift her down or lift her up, she can get furious when she feels that … when she doesn’t feel completely safe. Nothing works then, no matter what activity it is. Therefore, a lot is also on me, she is so confident that I know and parry her involuntary movements, but she does not have the same trust in the girls or the assistants who do not know her. It's hard to say what … but it's really more about the situation and the constellation that makes when it works and when it doesn’t. (Mother to child 7)

These people need to be familiar with the child and the child's individual interests and challenges, and in addition even have a great knowledge and understanding of the child's disability.

Yes, [they] need to have this in-depth knowledge of [her]. You should be able to read her if she thinks it's fun or if she thinks it's boring. You can make her think it's fun, yes, but you need to know her … as a person. (Mother to child 1)

Personal chemistry between the child and the people around were considered as crucial.

It is very important, it's the most important thing of all. That it is the right person. Otherwise, he becomes hysterical, he becomes sad, he doesn’t want to. He goes down, just like another person, right? He feels bad inside, you can see that right away after two minutes with a person. It's important for his safety … because he needs help, and then that's the most important thing, the assistant and those in the family. If it is the right person that he knows, it is personal chemistry. (Mother to child 10)

However, the parents found it very difficult to find these right people, which in turn increased the burden on the parents. The network around the child consisted of the parents and the right people, and when this network was well-functioning, the parents experienced that their child received the right support.

First of all, it depends on us adults around, because she can’t do anything without our help. So the number one is people nearby, assistants, us parents and maybe staff at school and so on  … then it is us all around who are number one. (Mother to child 12)

#### 3.2.2. Being dependent on an accessible environment

When the child wanted to participate in physical activities, the place where the activity was arranged was important. Long travel distances could mean that the child will not be able to attend. Parents or others were needed to transport the child and the travel time might be too tiring for the child. In addition, the accessibility of the place was crucial, and the changing rooms were often described as being too small and too crowded.

The [accessible] swimming pool is at the hospital. It is very narrow there and it always took very long time … we had to be there one hour early, and everyone had to share this changing room … it's time, you’re so pressed for time, it takes so long. There isn’t enough time … we always need to be two adults with him in the pool. (Father to child 10)

#### 3.2.3. Being dependent on access to assistive devices

The parents described children with CP who are non-ambulant as being dependent on access to assistive devices for participating in physical activities. However, the parent experienced that the right equipment in form of tailored assistive devices was difficult to find and get access to but necessary.

She does pretty well on her [adapted] bike as long as there are good streets, and not like uphill or a pothole or stuck on the curb. But otherwise, she can only cycle straight without any curves. (Father to child 8)

The assistive device Innowalk for dynamic standing exercise was perceived as easy to use and as the only possibility for the child to exercise regularly. The parents experienced that exercise in the Innowalk gave many positive health effects for their child.

She doesn’t have, she doesn’t experience pain, in her stomach as before. When she has exercised in the Innowalk, she eats and sleeps better. Because she moves herself, she feels happy and more alert, and she has energy. (Mother to child 5)

The device was placed in their home and the physical activity enabled through the Innowalk was experienced as energy- and time-saving for the child and the parents. Additionally, the right people were needed to support the child while being physically active in the Innowalk. It was important to find fun activities to do while exercising in the Innowalk, as listening and dancing to music, playing with toys, or talking with the people around them.

She can be really sweaty, like when she has exercised for half an hour in front of Just Dance, she's very, very warm. So she loves it. (Mother to child 7)

Those activities were used to divert the child or to just have fun while exercising in the Innowalk.

The walker is not as much fun as the Innowalk because she can … when she exercises [in the Innowalk] she can paint, play, yes. (Father to child 5)

The parents described their children as being involved in the dynamic standing exercise and they found the exercise meaningful. The children asked for the dynamic standing exercise, verbally, through body language, or alternative communication.

So she asks every day if she can go on the Innowalk; she may have gone in the morning and then she still drives forward [in her wheelchair] and then she says “that, that, that”, and she wants to go again. (Mother to child 7)

With the right support, the child could be independent in the Innowalk, which was not possible in other activities. The parents felt that the child was safe while exercising and they could step back for a while.

With [the] Innowalk I feel safe, and my daughter feels safe. We feel safe. (Father to child 5)

### 3.3. Getting hope in a challenging life situation

Living in a family with a child with a severe disability is challenging and both energy- and time-consuming. When support through people, environment, and assistive devices was lacking, the parents experienced being in a hopeless life situation. During the pandemic, many activities were cancelled, and some children did not attend school for longer periods. Getting access to the Innowalk, enabling dynamic standing on a regular basis, gave hope to the parents. The hope could be small or huge.

#### 3.3.1. Living near life and death

Parents said that the child's condition could quickly worsen and become life-threatening, for example, when the child got a cold or became ill. A small positive change of the health conditions meant a lot.

And her health has gotten a lot better, yes, because she was sick so much before. But when she exercises, the blood pressure is better. (Mother to child 5)

Her cough is better, not as much mucus. Before she had so much mucus. (Father to child 5)

He suffered from a life-threatening illness because of, uh, problems he had after the scoliosis surgery … he ended up in the hospital, in the intensive care unit. Right now, it's only the Innowalk he can do … we are isolated at home. And we are also waiting a while, not only because of Covid 19, this pandemic, but it is also his illness. We want to wait a while and he will stay at home as long as possible. (Mother to child 4)

#### 3.3.2. Getting hope

The parents experienced hope when their child could participate in physical activities.

Because it lengthens our, what can you say? … if we did nothing, then it will be that we lost our health, our energy, our hope. Quite simply, yes. It is something that gives us hope, quite simply, I say. When he does a physical activity, it feels that he has, how can you say … that he is, that he thinks it is good, that he lives, he lives. (Mother to child 4)

The families chose to participate in the study where their child could exercise in the Innowalk, and they believed that the device would help their child.

There is the Innowalk, if we could have had it when he was 7 years old, my son might be able to walk today, with just a little support. So it is fantastic, this machine. (Mother to child 4)

When exercising in the Innowalk, parents experienced their child being independent.

But it's just like helping him all the time. But we don’t do that when he pedals [exercises in the Innowalk] because he does that himself … It's not us, it's not us who force him and tell him to do things. He can stand and pedal by himself without someone being on him all the time. (Father to child 10)

Exercise in the Innowalk was experienced as, and expressed as being like, medicine with medical health benefits, and there was a great desire for a healthy future for their child.

[The Innowalk] strengthens the whole body, so that she is up and moving … Exercise will be another matter for her, but it can be … great for the entire airways and lungs and stretches and strengthens the entire body. …so that she can cope better when she gets colds and flus and things like that, so that it's a little more medical, maybe I’m thinking about her in the long term there. To better cope with future infections, simply. And then it's fun for her while it's going on, these moments she's more active give her joy. And of course, it's beneficial for her in the long run as well, but as I said, that is a little harder to see. (Mother to child 12)

## 4. Discussion

In this study, interviews with the parents of children with CP revealed that participation in physical activity was related to several positive outcomes of great value to both parents and children. In the everyday life of parents, physical activity was associated with a sense of immediate wellbeing and bodily feedback, and something that was strived for but sometimes difficult to integrate in a hectic schedule of work and caregiving duties. For parents, physical activity was also a source of recovery and building of resources to cope with their life situation. Beyond its immediate positive effects, physical activity was connected to the future and long-term health-related benefits. In the lifeworld of these families, physical activity can be interpreted as a source of strength and potential. It was seen as having a high intrinsic value in the moment in that it provided positive bodily feedback, and through long-term effects, it was also a bridge to the future. For the parents, physical activity had intrinsic value at the same time as it was an investment in the future, and this reasoning was applied to their children's physical activity as well.

Participation in physical activity was something that brought together the parent and child and had the potential to provide a sense of purpose and direction in connecting the present with the future in a meaningful and hopeful way. It was also a part of the life of these families that highlighted what parents and children had in common with each other and what their children had in common with other children—such as the bodily pleasures of swimming, riding, or being able to move. These findings illustrate the multiple values realized by physical activity in spite of the challenges this group of patients and families encounter. The findings broaden the understanding of what the right to physical activity and current recommendations can mean for these children and their caretakers (4, 5). Participation in physical activity implies more than just attending an activity, the person being physically active also needs to be involved. Involvement includes elements of motivation, persistence, social connection, and affect (26). The parents in our study experienced these factors as crucial, and they told of many ways for their child to be involved in the dynamic standing exercise. The children wanted to exercise and asked for the Innowalk, they wanted to listen and dance to music while exercising, or to interact with people around them. The parents were aware that it made no sense to exercise when the child did not want to. Thus, the parents experienced that their children went from hardly any physical activity to being able to be physically active. There was no other possibility for their child to be physically active on a daily basis and for a longer time period. The parents described the Innowalk as an investment for a healthy future as possible. However, they were also aware of the higher costs of the Innowalk compared to static standers, which is in agreement with our findings in an earlier study (20). Another way of being physically active for people with moderate-to-severe walking impairment is Frame Running, allowing them to propel themselves using a three-wheeled frame with a saddle and handlebars. Frame Running has shown to be a safe activity with benefits on physical fitness, functional mobility, and psychosocial outcomes (27). However, the participants in our study were not able to perform Frame Running at all or on a daily basis due to their severe physical disability. The Lokomat is another assistive, and robotic, device for people with moderate-to-severe walking impairment. The patient's leg movements can actively be guided while walking on a treadmill through an electrically powered, computer-controlled lower-limb exoskeleton. The Lokomat shows some inconsistent effects on walking abilities in three studies, and it is used in a therapeutic setting at rehabilitation centers and not in a home setting (28).

However, the children's physical activity needed to be supported by assistive devices, the child's network, consisting of family and other important adults as personal or school assistants, and accessible environments. The children were dependent on others for their physical activity and the parents were, in turn, also dependent on others to be able to support their children. In the lifeworld of these parents, dependence was a recurrent theme. Though participation in physical activity is something that most actors want to contribute to, the heavy dependence on other people and supporting conditions (as transport, assistive devices, and facilities) can make it difficult to achieve to the extent wished for. These findings add to the understanding of the complexity of supporting participation in physical activity in non-ambulant children with CP. Implementation of training regimes and any new methods or assistive devices has to be informed by an awareness of the embeddedness of the child in a network of relations. Our findings, therefore, illustrate why it is important to consider the complexity outlined by Skivington et al. (22). To the extent that dependence becomes a dominant feature in how the families give meaning to their situation, it also has consequences for relations with health professionals, personal assistants, and school. In our study, the parents expressed how dependent they felt on others, on an accessible environment, and on the access to tailored assistive devices. Our findings are in concordance with the findings of Abid et al. (29) that personal and environmental factors influencing physical activity behaviors among youths living with CP are complex and that they interact with each other. As a consequence, a person-based approach for rehabilitation interventions is needed (29). Interaction that is perceived to increase a sense of dependence runs the risk of undermining the potential for autonomy and empowerment. This might be experienced not necessarily as passivity but often takes the form of a troubled relation with special demands, criticisms, and sometimes unrealistic expectations. On the other hand, conditions and relations that reduce a sense of dependence can have profound consequences for the families (22). An example of this was the positive experience of having access to an assistive device for physical activity in the home providing flexibility and easy access—something that was of particularly high value during the Covid-19 pandemic when many children were restricted to their homes. The opportunity for physical activity at home is important for people with an immunocompromised status. People with advanced cancer perceived that the Covid-19 pandemic changed everything and that they could not be physically active at a fitness center because they did not dare to leave their homes or were restricted to their homes due to lockdowns (30).

Physical activity was for most of the parents related to hope in one way or another. It could be small, everyday hopes about that things will work out and that their children would enjoy the experience of an activity, but it could also be bigger hopes: hopes about cures, or that the right device may help their children in new and better ways. Hope has been found to be of great importance for coping with chronic illness (31) and refers to “… the possibility of an individual to take charge of his existence, to give it direction and to transcend the simple material aspects of reality. In addition, hope exists because we are relational beings, called to be in communion with others” (31, p, 105). Hope can thus be conceptualized as a phenomenon that helps the individual to bridge the current challenging situation with a better situation in the future. Hope can thus be seen as a kind of psychological capital (32) that is crucial for dealing with adversity and is thus related to goals, planning, and problem-oriented coping styles. Hope is also a controversial concept in that realistic hope in spinal cord injury has been shown to have a strong association with better coping, improved life satisfaction, and less depression, while unrealistic hope can have a negative impact on rehabilitation (33).

Hope for the families in this study may have more in common with findings from studies on chronic or degenerative conditions where hope seems to transform over time as the condition or circumstances change (31). Our findings show that hope is part of the total lifeworld of these families and is a dynamic element interlaced with the investment in physical activity and the matrix of social and material conditions that either support dependence or autonomy. A strong presence of dependence will thus have implications for the nature of hope. Concerns about the risk related to unrealistic hope and the so-called therapeutic misconception (false beliefs guiding the intent to take part in a clinical trial) (34) should be considered in the holistic appreciation of the child's and the family's situation. High dependence on, and difficult relations with, healthcare professionals and other actors may increase the risk of unrealistic hope while a certain degree of autonomy and empowerment will allow for more realistic hope and the possibility to draw on hope as source for coping. Interventions that support autonomy and limit the negative consequences of dependency might be beneficial in themselves and can also contribute to strengthen the constructive aspects of hope. To advance our understanding of the lived experience of phenomenon physical activity among parents to children who are non-ambulant, there is a need for more research involving other stakeholders as healthcare professionals, managers, politicians, and researchers and combining different theories, to arrive at a fuller understanding of the challenges of implementing new strategies to support participation in physical activity.

We had in this interview study a limited number of participants, which might be seen as a limitation. However, the participants had various lived experiences and we could perform in-depth analyses. It would be interesting in a future study to ask the children themselves about their lived experiences.

## 5. Conclusions

Physical activity has both immediate and long-term positive effects. It can also help support autonomy and is a source of strength for families. It is thus of vital importance to attend to physical activity recommendations and use the generative potential of participation in physical activity in work with families. Participation in physical activity for children with CP who are non-ambulant can be possible through an elaborate network of social relations and environmental conditions. To support physical activity, it is vital to limit the degree of dependence and contain the negative consequences of a high degree of dependence. Relations, support, and assistive devices such as the Innowalk that strengthen empowerment and autonomy should be prioritized, and if this works, the experience of physical activity can be positive, giving families hope. Physical activity is closely related to hopes for the child's future health and wellbeing. The degree to which hopes are realistic and helpful is part of the general outlook and lifeworld of the families. We suggest that the degree of dependence and the quality of relations is important for understanding the role of hope and its consequences for these families.

## Data Availability

The datasets presented in this article are not readily available because the data used in this study include sensitive information about the study participants, who did not provide consent for public data sharing. The current approval by the Regional Ethical Review Board in Lund, Sweden (2019-00106), does not include data sharing. A minimal data set could be shared by request from a qualified academic investigator for the sole purpose of replicating the present study, provided the data transfer is in agreement with EU legislation on the general data protection regulation and approval by the Swedish Ethical Review Authority. Requests to access the datasets should be directed to ÅBT, asa.tornberg@med.lu.se.
